# Investigation of herb-drug interactions with *ginkgo biloba* in women receiving hormonal treatment for early breast cancer

**DOI:** 10.1186/2193-1801-2-126

**Published:** 2013-03-22

**Authors:** Janette Vardy, Haryana M Dhillon, Stephen J Clarke, Inger Olesen, Felicity Leslie, Anne Warby, Jane Beith, Anne Sullivan, Anne Hamilton, Philip Beale, Anneliese Rittau, Andrew J McLachlan

**Affiliations:** 1 Sydney Medical School, University of Sydney, Sydney, NSW 2006 Australia; 2 Sydney Cancer Centre, Royal Prince Alfred and Concord Hospitals, Sydney, Australia; 3 Centre for Medical Psychology and Evidence-based Decision Making, University of Sydney, Sydney, Australia; 4 Psycho-oncology Co-operative Research Group, University of Sydney, Sydney, Australia; 5 Faculty of Pharmacy, University of Sydney, Sydney, NSW Australia; 6 Centre for Education and Research on Ageing, Concord Hospital, Concord, NSW Australia

**Keywords:** Anastrozole, *Ginkgo biloba*, Herb-drug interaction, Letrozole, Tamoxifen

## Abstract

Women receiving treatment for breast cancer commonly ingest herbal medicines. Little is known about the potential for herb-drug interactions in this population. The aim of this study is to investigate the effect of *ginkgo biloba* co-administration on the pharmacokinetics of tamoxifen, anastrozole and letrozole. This was a prospective open-label cross-over study in 60 women with early stage breast cancer taking either tamoxifen, anastrozole or letrozole (n=20/group). Participants received *ginkgo biloba* (EGb 761) for 3 weeks (120 mg twice daily). Trough concentrations of drugs were measured before and after *ginkgo biloba* treatment using LC-MS/MS. Toxicities were graded according to National Cancer Institute Common Terminology Criteria for Adverse Events. Trough concentrations before and after treatment with *ginkgo biloba* were not significantly different for tamoxifen (93.5 ± 29.0, 86.5 ± 25.3 ng/mL; p=0.16), letrozole (91.1 ± 50.4, 89.6 ± 52.14 ng/mL; p=0.60) or anastrozole (29.1 ± 8.6, 29.1 ± 7.6 ng/mL; p=0.97). *Ginkgo biloba* was well tolerated, with no difference in toxicity during *ginkgo biloba.* Co-administration of *ginkgo biloba* does not significantly affect the pharmacokinetics of tamoxifen, anastrozole or letrozole. There was no difference in the toxicity profile of hormone therapy with *ginkgo biloba* use in women with early stage breast cancer.

## Introduction

Complementary and alternative medicines (CAM) are widely used by a large proportion of cancer patients (mean 36%, range up to 80%) (Sparreboom et al. 2004). It is estimated that at least 50% of patients use herbal supplements and/or anti-oxidants at some time during their cancer journey (Adler & Fosket 1999; Roberts et al. 2005). The highest use of CAM in people with cancer is in women (Tascilar et al. 2006), those with breast cancer (Gerber et al. 2006), of younger age (Tascilar et al. 2006; Bennett et al. 2009), with higher levels of education (Steinsbekk et al. 2000), more advanced disease and of Asian ancestry. The majority of patients do not inform their oncologists or medical practitioners that they are using CAM (Adler & Fosket 1999; Roberts et al. 2005; Richardson et al. 2004). An Australian study found that 55% of patients using biologically based CAM and 80% of those using non-biologically based CAM did not discuss their use of CAM with their oncologist (Kremser et al. 2008). However, relatively limited evidence is available to guide clinicians and consumers on the clinical significance of interactions between CAM and medicines used in the treatment and prevention of cancer (Sparreboom et al. 2004).

The herbal medicine *ginkgo biloba* is commonly used in the community (Sparreboom et al. 2004). EGb 761 is a standardised concentrated extract of *ginkgo biloba*, containing 24% flavone glycosides and 6% terpene lactones and is produced from ground ginkgo leaves (Braun 2007). It is registered as a prescription medicine in France and Germany for the treatment of deficient memory, disturbances of concentration, depressive mood, dizziness, tinnitus and headache (product information sheet) (Schwabe 2004). Multiple randomised controlled trials (RCT) have shown no difference in side effects between patients allocated to *ginkgo biloba* or placebo (Le Bars et al. 2000; DeKosky et al. 2008).

No published RCT have assessed the effects of *ginkgo biloba* on anti-cancer hormone pharmacokinetics (Sparreboom et al. 2004), but there is indirect evidence to suggest that *ginkgo biloba* does not significantly affect drug metabolising enzymes responsible for the metabolism of chemotherapy agents and tamoxifen (Coxeter et al. 2004; Coxeter et al. 2003a). Our previous research investigated the effects of *ginkgo biloba* on warfarin in healthy subjects (metabolised by the enzyme CYP2C9) (Jiang et al. 2005) and two other studies have demonstrated a lack of effect of *ginkgo biloba* on the drug metabolising enzymes CYP3A4, CYP2D6, CYP1A2 and CYP2E1 and CYP2C9 in healthy volunteers (Markowitz et al. 2003; Gurley et al. 2002).

The aim of this study is to investigate the effect of *ginkgo biloba* co-administration on the pharmacokinetics of tamoxifen, anastrozole and letrozole in women with early stage breast cancer.

## Methods

### Materials

Anastrozole, D12-anastrozole, letrozole, D4-letrozole, tamoxifen and D5-tamoxifen were purchased from Research Chemical North York (Ontario, Canada). Methanol was purchased from Fisher Scientific (New Jersey USA). Orthophosphoric Acid (85%), hydrochloric acid (AnalaR) and ammonia (AR) were purchased from BDH, Biolab (Scoresby, VIC, Australia). Formic Acid (AR grade) was purchased from Merck (Kilsyth, VIC, Australia).

Strata-X-C 33Hm Cation Mixed-Mode Polymeric Sorbent solid phase extraction cartridges (200 mg /3 mL) were obtained from Phenomenex (Torrana, CA, USA).

### Pharmacokinetic study

This was a prospective open-label cross-over study in 60 women with early stage breast cancer taking tamoxifen, anastrozole or letrozole (n=20/group). Women were being treated at Concord Repatriation General Hospital, Royal Prince Alfred Hospital and Strathfield Breast Centre in Sydney, Australia. All women had previously completed surgery ± adjuvant chemotherapy for localised breast cancer, and had no evidence of a recurrence of their cancer. They had to have been on a stable dose of the hormone for a minimum of two months, to ensure steady-state had been achieved. The study exclusion criteria included: use of trazodone, monoamine oxidase inhibitors, thiazide diuretics, anticoagulation, or anti-platelet medications other than low-dose aspirin or coumarin/heparin flushes associated with use of a portacath, and ingestion of ginkgo biloba within 4 weeks of the study commencement.

At baseline, toxicity data for hormone treatment and *ginkgo biloba* were collected. Women then commenced an open label standardised extract of *ginkgo biloba* (EGb 761, 120 mg bd) for a three week period. Toxicity data were collected at the end of the dosing interval. Plasma samples to measure the concentration of the anastrozole, letrozole or tamoxifen were taken to assess trough concentrations of each drug.

The study was approved by the Human Research Ethics Committee of all participating institutions and written informed consent was obtained from all participants.

### Sample preparation and liquid chromatography tandem mass spectrometry analysis

Blood samples were collected into heparinised collecting tubes by venepuncture. Samples were centrifuged for 10 min at 4°C and plasma transferred into vials, which were stored at -70°C until analysed. Sample preparation was based on the publication by (Beer et al. 2010). In short, plasma samples (200 μL) were spiked with the appropriate deuterated analogue internal standard (10 μL D12-anastrozole, 10 μL D4-letrozole or 20 μL D5-tamoxifen), water (1 mL) and 2% phosphoric acid (40 μL) and then extracted using solid phase extraction cartridges. The SPE cartridges were conditioned with methanol (2 mL) followed by 0.2% phosphoric acid (v/v, 2 mL). The plasma samples were allowed to pass through under gravity and then the SPE cartridges were washed with 0.2% phosphoric acid (3 mL), 0.1 M hydrochloric acid (2 mL) and methanol (2 mL). The cartridges were then dried under vacuum and the target analytes were eluted with 5% ammonia in methanol. The eluate was evaporated to dryness and reconstituted in 30 μL of methanol and 270 μL of 0.1% formic acid. A 5 μL aliquot was injected into the liquid chromatography tandem mass spectrometry (LC-MS/MS) system for analysis.

The chromatographic system consisted of a Waters Acquity® UPLC solvent delivery system and autosampler (Milford MA, USA) and an AB Sciex QTrap 4000 linear quadrupole mass spectrometer (Concord, ON, Canada) equipped with a turbo ion spray (ESI) source. The mass spectrometer was operated in positive ion mode. Multiple reaction monitoring (MRM) was performed using specific precursor-to-product ion transitions m/z. Chromatographic separation was accomplished on a Restek PFP C_18_ HPLC column (50 mm X 2.1 mm ID, 3 μm, 100 mm x 2.1 mm). The mobile phase consisted of 0.1% formic acid (solvent A) and 0.1% formic acid in methanol (solvent B) with a flow rate of 0.2 mL/min.

The calibration curves for each analyte were linear over the concentration range of 2.5 to 750 ng/mL. The lower limit of quantitation for anastrozole and letrozole was 2.5 ng/mL and 10 ng/mL for tamoxifen. The extraction recovery from human plasma was considered acceptable with a mean recovery of greater than 88% for each analyte. Minimal ionization suppression was observed in both the low concentration and high concentration spiked plasma samples and the variation in matrix effects between the five different sources of plasma was minimal for all analytes over a range of concentrations. No endogenous peaks were identified in the extracted ion chromatograms that would cause interference with drug quantitation.

The assay performance was assessed over 3 days (4 replicates) at 3 concentration levels. The relative error for each analyte was less than 9% and the intra-day and inter-day precision (%RSD) was less than 7% for anastrozole, letrozole and tamoxifen.

### Data analysis

Steady-state trough concentrations of each drug were compared before and during treatment with *ginkgo biloba* extract. Comparisons were made using a paired t-test. Toxicities are graded according to National Cancer Institute Common Terminology Criteria for Adverse Events Version 3.0 (NCI CTCAE).

## Results

Sixty women with early stage breast cancer participated; 20 were using tamoxifen, letrozole and anastrozole respectively. The 48 (80%) women who had received adjuvant chemotherapy were a mean of 37 months (range 4–113) from completion of chemotherapy. Twenty four (40%) had received a taxane-containing regimen and 24 (40%) an anthracycline regimen, with 6 (10%) women also being treated with adjuvant Trastuzumab. Twelve (20%) women had not required adjuvant chemotherapy. The median age of participants was 57 years (range: 33– 86 years).

**Figure 1:**
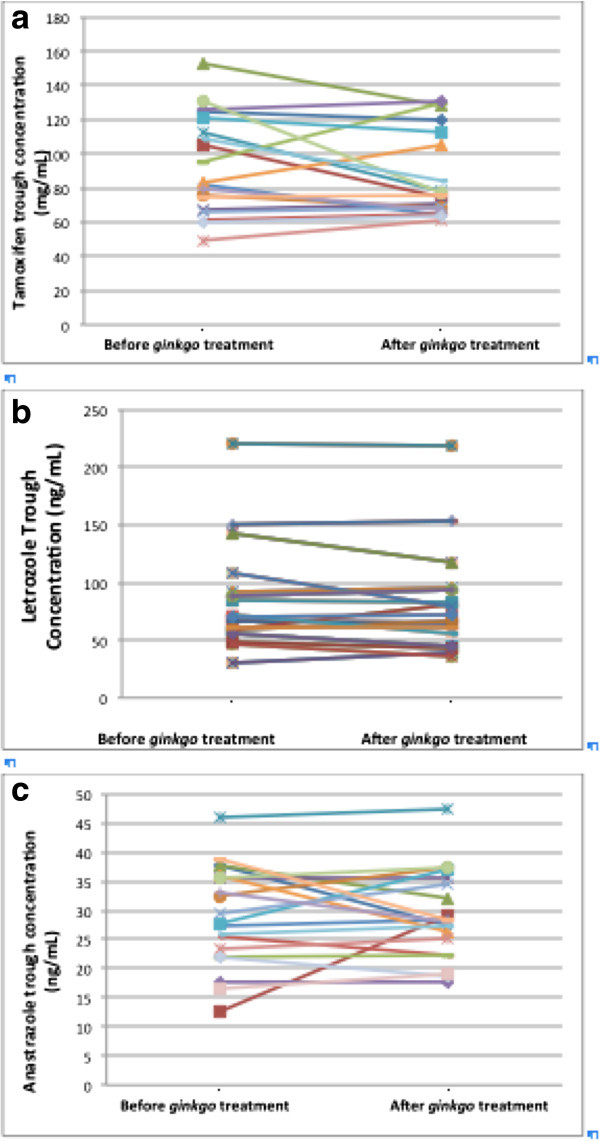
**Trough concentration of anti-estrogenic agents with and without*ginkgo biloba*(GB) treatment. A**) Tamoxifen. **B**) Letrozole. **C**) Anastrazole.

Individual trough concentrations of the anti-estrogenic agents prior to using *ginkgo biloba* and after 3 weeks of *ginkgo biloba* treatment are outlined in Figure 1. There was no significant difference in the trough concentrations measured before and after treatment with *ginkgo biloba* for any of the three hormone treatments (Table 1).

**Table 1:** **Pharmacokinetics of tamoxifen, letrozole and anastrozole before and after treatment with*ginkgo biloba (GB)***

Drug	Drug trough concentration (ng/mL) before ***GB*** treatment	Drug trough concentration (ng/mL) during ***GB*** treatment	P-value*
Tamoxifen (*N*=20)	93.5 ± 29.0	86.5 ± 25.3	*P* = 0.16
Letrozole (*N* =20)	91.1 ± 50.4	89.6 ± 52.14	*P* = 0.60
Anastrozole (*N* =20)	29.1 ± 8.6	29.1 ±7.6	*P* =0.97

*paired test; data presented as mean ± standard deviation.

Thirty-one of the 60 women reported no side effects. There were no grade 3 toxicities but two women reported grade 2 toxicities (1 headache, 1 flu-like symptoms). The most common side effects were headache (n=8), flatulence (n=6), hot flushes (n=5), nausea (n=2) and diarrhoea (n=2). One woman each complained of appetite loss, dizziness, disturbed dreams, dry skin, flu-like symptoms, pain in extremities and a metallic taste.

## Discussion

Although *ginkgo biloba* is a CAM that is commonly used by cancer patients, there is a lack of information about the impact of *ginkgo biloba* administration on cancer treatments such as chemotherapy and hormonal treatment. This study investigated the interaction between the anti-cancer hormonal agents tamoxifen, anastrozole and letrozole, and a standardised extract of *ginkgo biloba* (EGb761) in women with breast cancer. This patient population was selected as women with breast cancer are reported to use more CAM than other cancer patients, and the majority take tamoxifen, anastrozole or letrozole for a period of 5 years. It is therefore critical to know if there is a pharmacokinetic interaction between *ginkgo biloba* and common anti-cancer hormonal treatment.

There are no published controlled trials that have assessed the effects of *ginkgo biloba* on chemotherapy pharmacokinetics (Sparreboom et al. 2004), but there is indirect evidence to suggest that *ginkgo biloba* does not significantly affect drug metabolising enzymes responsible for the metabolism of chemotherapy agents and tamoxifen (Coxeter et al. 2004; Coxeter et al. 2003b). Our previous research investigated the effects of *ginkgo biloba* on warfarin in healthy subjects (metabolised by the enzyme CYP2C9) (Jiang et al. 2005) and two other studies have demonstrated a lack of effect of *ginkgo biloba* on the drug metabolising enzymes CYP3A4, CYP2D6, CYP1A2 and CYP2E1 and CYP2C9 in healthy volunteers (Markowitz et al. 2003; Gurley et al. 2002). While there is some *in vitro* cell evidence that selected phytoconstituents of ginkgo have potentially estrogenic (Oh & Chung 2004) and antiestrogenic effects (Oh & Chung 2006) this has not been supported by evidence from clinical studies in patients using recommended orally administered doses of a standardised extract of *ginkgo biloba* (EGb 761). The putative anti-estrogenic effects observed in vitro are not likely to have effects on estrogen therapy in people (Oh & Chung 2006).

Zadoyan et al. recently reported a cocktail drug interaction study exploring potential interaction with the *ginkgo biloba* standardised extract EGb 761 (Zadoyan et al. 2012). This study in 18 healthy volunteers found that EGb761 had no relevant effect on the metabolic activity of the major drug metabolizing CYP enzymes. The authors concluded that *in vivo* EGb761 treatment is not likely to cause clinically relevant metabolic drug-drug interactions.

Several large placebo-controlled RCT have found *ginkgo biloba* to be extremely well tolerated, with no differences in side effects between those receiving *ginkgo biloba* and those receiving placebo. In our study the majority of participants reported either no side effects or minor side effects such as headache, flatulence and hot flushes - some of which are likely to have been due to the hormonal treatment rather than the *ginkgo biloba*.

In conclusion, *ginkgo biloba* (EGb761) co-administration did not significantly affect the pharmacokinetics of tamoxifen, anastrozole or letrozole, and was well tolerated with minimal side effects. Our results suggest that the co-administration of EGb761 is unlikely to lead to clinically significant interactions for women who are receiving hormonal treatment for breast cancer. A study is in progress evaluating the pharmacokinetic impact of *ginkgo biloba* on common chemotherapy agents in women receiving adjuvant chemotherapy for early stage breast cancer.
